# A cross-sectional mixed-methods study of sexual and reproductive health knowledge, experiences and access to services among refugee adolescent girls in the Nakivale refugee settlement, Uganda

**DOI:** 10.1186/s12978-019-0698-5

**Published:** 2019-03-19

**Authors:** Olena Ivanova, Masna Rai, Wendo Mlahagwa, Jackline Tumuhairwe, Abhishek Bakuli, Viola N. Nyakato, Elizabeth Kemigisha

**Affiliations:** 1 Division of Infectious Diseases and Tropical Medicine, Medical Centre of the University of Munich (LMU), 80802 Munich, Germany; 2 Faculty of Interdisciplinary Studies, Mbarara University of Science and Technology, P.O. Box 1410, Mbarara, Uganda

**Keywords:** Adolescent, Girls, Refugee, Migrants, Sexual and reproductive health, Experiences, Access, Africa

## Abstract

**Abstract:**

**Background:**

Humanitarian crises and migration make girls and women more vulnerable to poor sexual and reproductive health (SRH) outcomes. Nevertheless, there is still a dearth of information on SRH outcomes and access to SRH services among refugee girls and young women in Africa. We conducted a mixed-methods study to assess SRH experiences, knowledge and access to services of refugee girls in the Nakivale settlement, Uganda.

**Methods:**

A cross-sectional survey among 260 adolescent girls 13–19 years old was conducted between March and May 2018. Concurrently, in-depth interviews were conducted among a subset of 28 adolescents. For both methods, information was collected regarding SRH knowledge, experiences and access to services and commodities. The questionnaire was entered directly on the tablets using the Magpi® app. Descriptive statistical analysis and multinomial logistic regression were performed. Qualitative data was transcribed and analysed using thematic content analysis.

**Results:**

A total of 260 participants were interviewed, with a median age of 15.9 years. The majority of girls were born in DR Congo and Burundi. Of the 93% of girls who had experienced menstruation, 43% had ever missed school due to menstruation. Regarding SRH knowledge, a total of 11.7% were not aware of how HIV is prevented, 15.7% did not know any STI and 13.8% were not familiar with any method to prevent pregnancy. A total of 30 girls from 260 were sexually active, of which 11 had experienced forced sexual intercourse. The latter occurred during conflict, in transit or within the camp. A total of 27 of 260 participants had undergone female genital mutilation (FGM). The most preferred sources for SRH information was parents or guardians, although participants expressed that they were afraid or shy to discuss other sexuality topics apart from menstruation with parents. A total of 30% of the female adolescents had ever visited a SRH service centre, mostly to test for HIV and to seek medical aid for menstrual problems.

**Conclusions:**

Adolescent refugee girls lack adequate SRH information, experience poor SRH outcomes including school absence due to menstruation, sexual violence and FGM. Comprehensive SRH services including sexuality education, barrier-free access to SRH services and parental involvement are recommended.

**Electronic supplementary material:**

The online version of this article (10.1186/s12978-019-0698-5) contains supplementary material, which is available to authorized users.

## Plain English summary

Although it is known that humanitarian crisis makes girls more vulnerable to unwanted pregnancies, HIV, maternal death and sexual violence, there is still a dearth of research on sexual and reproductive health (SRH) outcomes and access to SRH services among refugee girls in Africa. We conducted a study to assess SRH experiences and knowledge of refugee girls in the Nakivale settlement in Uganda using quantitative and qualitative methods. A total of 260 girls aged 13–19 participated in the survey and 28 of them took part in the in-depth interviews. Data analysis showed that many girls lacked knowledge about sexually transmitted diseases including HIV and contraception. A total of 27 of 260 girls had undergone female genital mutilation. Among 30 sexually active girls, 11 had experienced forced sexual intercourse which occurred during conflict, in transit or in the camp. The most preferred sources for SRH information were parents or guardians, although girls shared that they were afraid or shy to discuss other sexuality topics with parents apart from menstruation. In conclusion, our study showed that refugee girls lack adequate SRH information and experience poor SRH outcomes including school absence due to menstruation and sexual violence. Comprehensive SRH services including sexuality education, barrier-free access to SRH services and parental involvement are recommended in order to improve their SRH knowledge and outcomes.

## Background

In 2018, there were 68.5 million people in the world who were forced to leave their homes, and among them, 25.4 million became refugees [1]. Crisis increases the vulnerability of women and girls to unwanted pregnancies, HIV and sexually transmitted infections (STIs), maternal death and sexual violence. Out of the 830 women and adolescent girls who die every day due to complications during pregnancy and childbirth globally, 507 are those from the displaced population [2]. Refugees and migrants are considered to be at high risk of sexual victimization [3–5].

The unprecedented fact is that more than half of the refugee population are under the age of 18 [1]. Despite these numbers, there is no satisfactory prioritization of sexual and reproductive health (SRH) challenges faced by the adolescents (10–19 years old) in humanitarian settings, and their SRH needs are often neglected. For instance, knowledge of refugee girls in African countries regarding contraceptive methods, STIs and HIV/AIDS is limited [6]. This population often has limited access and availability of SRH services due to distance, costs and stigma [6].

Uganda, ranked as Africa’s largest and the world’s third largest refugee hosting country after Ethiopia and Kenya, is currently home to 1,252,470 refugees and asylum seekers [7]. Twenty-seven percent of these refugee populations are girls under the age of 18 [8]. As a hosting country, Uganda has the youngest population in the world with a median age of 15.2 and adolescents comprise 24% of the population [9]. The median age at first sexual intercourse is 16.9 years [10] and the teenage pregnancy rate in Uganda is 25%, one of the highest in Sub-Saharan Africa [9]. The knowledge and use of contraceptives seem to be limited in this age group. The modern contraceptive prevalence among women age 15–24 is 21.8% [11]. Seventy-five percent of unmarried women aged 15–19 reported having received gifts or money in exchange for sex [12]. However, there is a limited body of evidence on the SRH of refugee girls living in Uganda. Women’s Refugee Commission and UNHCR report (2011) showed that girls in the Nakivale refugee settlement reportedly become sexually active at a very young age; often exchange sex for money starting by age 10, in some instances without having access to any family planning methods [13]. According to this report, there were ﻿four health facilities operating in the settlement, all of them provided some family planning methods. Staff was trained in these facilities, however reported some gaps in skills regarding long-acting contraception methods and often stock-outs. Youth-friendly services were absent, and girls were afraid to be seen and judged while visiting the maternity wing of a health facility [13].

This study aims to fill the knowledge gap and provide an overview of the situation on sexual and reproductive health experiences, knowledge and access to services among adolescent refugee girls living in a humanitarian setting in Uganda.

## Methods

### Study setting

Nakivale refugee settlement is located in Isingiro District in Southwest Uganda. It is the oldest and largest settlement in Uganda, currently hosting a diverse refugee population of more than 113,000 refugees. It was established in 1962 for refugees from Burundi and became predominantly Rwandese in 1994. This was followed by a large influx of Congolese refugees and a dramatic increase in the Somali population in the 1990s [13]. Women and girls comprise almost half (46.8%) of Nakivale’s total population and half of the population (50.2%) are below 18 years of age [14]. Many children and youth in Nakivale do not attend school, according to UNHCR household survey 54% of households with school aged children have at least one child not enrolled in school [15]. Main barriers to education are high school fees for secondary school, overcrowding and long travel distances [16]. There is limited access to water sources in the camp. Scares environmental resources cause tensions between refugees and the host community, for example, ccollecting firewood for cooking outside of the settlement puts women and girls at risk of sexual violence [16].

### Study design

We selected a mixed-methods design for this study, linking common themes across the survey and semi-structured interviews in order to facilitate comparison of qualitative and quantitative data. Data were collected from March to May of 2018.

#### Quantitative data collection and analysis

A total of 260 girls were enrolled in the quantitative study. The selection criteria for respondents were age between 13 and 19 years old, willingness to participate in the study, informed consent from parent/caregiver and assent for minors. Unaccompanied adolescents below 18 years of age (without a parent or a legal caregiver who could provide an informed consent) were excluded from the study. Respondents comprised a convenience sample of girls recruited in the different camp communities, e.g. Burundi or Congolese communities, and schools with the help of local community mobilizers and teachers.

The questionnaire consisted of four sections: demographic characteristics, e.g. age, country of origin and education; SRH knowledge, e.g. ways of HIV transmission and prevention, list of contraception methods (modern and traditional) and STIs; SRH experiences, e.g. forced intercourse, FGMs and pregnancy; and access to SRH services. Validated tools were used to develop this questionnaire - Reproductive Health Assessment Toolkit for Conflict-Affected Women, CDC, 2007 [17] and Adolescent Sexual and Reproductive Health Toolkit for Humanitarian Settings, UNFPA and Save the Children, 2009 [18].

A research team of four field workers was trained in research ethics and administering of the structured questionnaire. Data were collected using the tablets in a private setting without parents or caregivers been present. The questionnaire was programmed using the Magpi® application. It allowed for a data collection in an off-line mode. The completed questionnaires were uploaded directly to the central database when connected to the internet and immediately extracted in Excel format.

The statistical package - STATA version 14 and R 3.5.1 were used for the analysis. We performed descriptive statistics to present the findings from the questionnaire. We also evaluated the knowledge on SRH through four question-based components namely, 1- “Ways of HIV transmission”, 2- “Methods of HIV prevention”, 3- “Knowledge of STIs” and 4- “Knowledge of contraception”. Each of them was evaluated on an ordinal scale increasing from zero to three. Summary statistics on these outcomes have been described in the results for the observed data. We also combined these four components to obtain a univariate unweighted sum describing overall knowledge about SRH. This outcome of overall knowledge would range between zero and 12, on the ordinal scale. We categorized this outcome to low (average score up to one across four components), medium (average score between one and two across four components) and high (average score two or more across four components) levels for evaluation of individual explanatory variables (risk factors or covariates e.g. age, education) using a multinomial logistic regression. This method allowed us to evaluate the differential association of the individual candidate risk factors, and the outcome levels high and medium compared to low describing the score for overall SRH knowledge. Relative risk ratios (RRRs) describe this association outcome. RRRs are similar to the outcomes observed in a simple logistic regression; however, the above method allows us to joint comparison across more than two categories, which is an advantage over using simple logistic regression [19].

#### Qualitative data collection and analysis

Of the 260 girls who answered the questionnaire, 28 girls were purposively selected for the qualitative interviews to broadly represent different countries and age groups. Five interviews were excluded from the analysis because of a low quality, e.g. very short or unclear. The interviews were performed in English, Swahili or Runyankole by four field workers who were proficient in these languages. A guide for semi-structured interviews had been developed to guide the interviews but it allowed for flexibility. The guide was based on the topics addressed in the questionnaire and adapted to different age groups. For instance, young girls who were not sexually active yet discussed menstruation experiences, relationships with friends and family, SRH information sources etc. Same field workers who administered questionnaire, later performed in-depth interviews, which helped to build the trustful relationships with the participants. Interviews were performed in a private environment, e.g. in the classroom during the break when other children were outside, without a parent/caregiver/teacher been present. During the interviews, notes were taken and field workers checked them to ensure accuracy of the records. The interviews were recorded with voice recorders and transcribed. Interview transcripts were translated to English where applicable. Data were coded manually using colour coding and, primarily, deductive coding was applied. Two researchers reviewed all the transcripts and generated a codebook and themes based on the literature, our quantitative data and qualitative guide. We employed thematic content analysis for this research [20]. The findings are presented with original supporting source quotations from the 23 participants.

## Results

### Demographics characteristics of the participants

Two hundred and sixty girls participated in the survey and of them twenty-three girls participated in the interviews as well. The mean age of participants was 15.9 (SD 0.11, 95% CI 15.6, 16.0). The highest number of the girls were born in DR Congo (29.6%) and the lowest number came from Kenya, with only 1 participant out of the 260. The majority (about 37%) of the participants had been living in Uganda for more than 5 years or between 1 and 3 years (28.9%). Few of them (0.7%) could not recall or relate the duration of stay in Uganda and also a minority of them (4.2%) were staying in Uganda for less than a year. Seventy-one percent of girls had completed at least primary education and about 26% of them had completed secondary education. A total of 63.5% of girls were also currently in school. The demographic characteristics of the participants has been presented in Table 1.

**Table 1:** Demographic characteristics of the survey respondents

	Number (*n* = 260)	Percentage
Age		
Younger girls (13–15 years old)	119	45.8
Older girls (16–19 years old)	141	54.2
Country of Origin		
DR Congo	77	29.6
Burundi	45	17.3
Rwanda	35	13.5
Ethiopia	33	12.6
Somalia	25	9.6
Uganda (refugee born in Uganda)	21	8.1
South Sudan	13	5.0
Tanzania	8	3.1
Eritrea	2	0.8
Kenya	1	0.4
Duration of Stay in Uganda		
Less than 1 year	11	4.2
1–3 years	75	28.8
3–5 years	60	23.2
More than 5 years	96	36.9
Since birth	16	6.1
Don’t know	2	0.8
Education		
Primary	185	71.1
Secondary	67	25.8
Never attended school	5	1.9
Tertiary	2	0.8
Adult learning	1	0.4
Religion		
Protestant	167	64.2
Catholic	45	17.3
Jehovah’s witness	32	12.2
Orthodox	8	3.1
Muslim	6	2.3
Traditional	2	0.8
Currently in School		
Yes	165	63.5
No	95	36.5

### Sexual and reproductive health knowledge among girls

We assessed the knowledge of SRH topics, e.g. ways of HIV transmission, STIs and family planning via survey. A total of 23 out of the 260 (8.8%) participants did not know about the ways of acquiring HIV and 30 of them did not know about the method of HIV prevention. Similarly, 41 (15.7%) of the participants could not name any of the STI’s and 36 (13.8%) did not know about a single method of contraception. Among the girls knowledgeable about HIV acquisition, sexual intercourse with infected person was the most popular reason (85.8% of them had chosen this option). In the same manner, 80.4% of girls selected abstinence as a way of preventing HIV transmission. The SRH knowledge of the girls described through the four components has been presented in Table 2.

**Table 2:** Sexual and reproductive health knowledge among girls

	Number (*n* = 260)	Percentage
Ways of HIV transmission		
0	23	8.8
1	73	28.0
2	145	55.7
3 and more ways	19	7.5
Methods of HIV prevention		
0	30	11.7
1	166	63.8
2	54	20.7
3 and more methods	10	3.8
Knowledge of STIs		
0	41	15.7
1	134	51.5
2	48	18.4
3 and more STIs	37	14.4
Knowledge of contraception		
0	36	13.8
1	115	44.4
2	69	26.5
3 and more methods	40	15.3

A multinomial regression analysis showed that age, education level and teachers/school as a main source of SRH information were associated with better SRH knowledge. Results are presented in the Additional file 1.

These quantitative findings were confirmed by individual interviews. With respect to the route of HIV transmission, the girls frequently talked about sexual intercourse and sharing of sharp objects with an HIV infected person as risk factors. The other routes, such as mother to child transmission or breastfeeding, were not much discussed by them:

“… *don’t have sex or play with someone who has it* [HIV]. *Because if you have sex with someone who has HIV, it can be passed on to you. And still if you fight with an infected person, you may scratch him or he may scratch and your blood can easily mix.* ”(15 years old).

Similar results were seen for the ways of HIV prevention. The majority of girls during the interviews mentioned abstinence as an important method for preventing HIV acquisition or pregnancy. For the girls that knew more than one way of prevention, the next most well-known method was by the use of condoms.

The knowledge of girls about STIs was limited to HIV and syphilis for a vast proportion (80 and 31% of girls respectively). Some of them also talked about Hepatitis B and *Neisseria Gonorrhoea*, however, the symptoms of STIs were not clear to the girls.

The contraception methods often mentioned by girls were abstinence and condom use. Other than these methods, injection and pills were mentioned by few girls who had knowledge about two or more contraceptive measures:

*“The methods of preventing pregnancy are like this: when you are still a student, you must abstain from sex. Then when you are a woman and you want to have sex without producing, you can use pills, injections and self-control.”* (17 years old).

Misconception about family planning methods was also present:

“*… I cannot use family planning methods because they are not good. They can affect life.”* (17 years old, not sexually active and has not used contraception yet).

### Menstrual hygiene and commodities

Out of the 93% of participants that had already experienced menstruation, the average age for the start of menstruation was found to be 13.4 years (95% CI, 13.2, 13.6, SD 0.7). While most of the girls began menstruation at either 13 or 14 years, one of them had at nine and some at 16. A total of 78% of menstruating girls had access to disposable pads (distributed by the UNHCR) and used them followed by cotton cloth (made from rags) - 18%. There was also one case of using plant leaves during menstruation.

The challenges in accessing menstrual hygiene products were also discussed in the interviews. The participants reported that the UNHCR distributed 5–6 packets of pads per women on an average of 6 months which sometimes even had to be shared with other female family members. When lacking pads, the adolescents used old clothes, and these were often reused after washing:

*“Sometimes when it’s not there* [pad], *we use clothes.”* (16 years old).

*“For me here, when I get money, I buy some pads and, when I fail to get money, I tear clothes and use them.”* (19 years old).

“*We hang them* [clothes] *there outside. And then sit around to keep them until they dry, or else other people will steal them.”* (16 years old).

A total of 43% of the menstruating girls missed school during their menstruation due to multiple reasons. The main reason was pains during menstruation – named by 74% of girls. The girls were also afraid of staining (22%) and some had no product to manage menstruation (16%). Minority of them (2.8%) were afraid of being teased and the same proportion were also prevented from attending schools because of religious barriers or social taboos related to menstruation. During the interviews, the participants were frequently mentioning that they missed schools mainly because they had either severe pains or bleeding during the menstruation. This statement is also supported by the fact that one of the most common reasons to access health facilities was to seek help for menstrual problems.

### Sources of information and access to SRH services

For many participants (38.5%), the most important source of SRH information was schools or teachers. The second most important source (34%) was parents or guardians. Television, internet, books or magazines and male relatives were not considered as an important source by any of the girls. Although radio was mentioned as an important source of information by few of them (1.5%), none of them preferred radio as a source of SRH information. The important and preferential sources of SRH information by the participants ordered by ranking has been elaborated in Table 3.

**Table 3:** Sources of information on sexual and reproductive health topics

	Main source *n*, %	Second main source *n*, %	Preferred source *n*, %
School/teachers	100 (38.4)	37 (14.2)	36 (13.8)
Parents/Guardians	93 (35.7)	89 (34.2)	100 (38.4)
Friends/peers	26 (10.0)	50 (19.2)	30 (11.5)
Doctor/Nurse	20 (7.6)	20 (7.6)	44 (16.9)
Other female relatives	6 (2.3)	21 (8.0)	18 (6.9)
Sister	5 (1.9)	20 (7.6)	11 (4.2)
Radio	4 (1.5)	2 (0.7)	0 (0.0)
Religious organisations	3 (1.1)	5 (1.9)	4 (1.5)
Brother	1 (0.3)	1 (0.3)	1 (0.3)
Neighbours (community)	1 (0.3)	5 (1.9)	3 (1.1)
Partner (boyfriend)	0 (0.0)	3 (1.1)	1 (0.3)
Organizations/seminars	0 (0.0)	4 (1.5)	3 (1.1)
TV	0 (0.0)	0 (0.0)	0 (0.0)
Internet	0 (0.0)	0 (0.0)	0 (0.0)
Books/Magazine	0 (0.0)	0 (0.0)	1 (0.3)
Male relatives	0 (0.0)	0 (0.0)	0 (0.0)
Any reliable person (whom they trust of having SRH knowledge)	0 (0.0)	0 (0.0)	7 (2.6)
Total	260	100%	

During the interviews, girls mentioned that even though their mothers were obviously the first and the closest person they could approach for sharing and discussing the SRH problems, they hesitated to do so. Some of them because of shame and some of them due to fear. The discussions with their mothers were limited to menstrual problems and its management. Mothers also frequently advised them to abstain from sex until they were married without discussing other topics, such as contraception. The participants stated that they sometimes discussed HIV and pregnancy with their friends:

“*I talk to my mother, of course, but mostly I don’t share with my mother because I feel shy. So, I have to ask friends who have experienced those things. I can consult them about how you do abortion, prevent HIV and ways through which one can acquire HIV. That’s what I ask them.”* (17 years old).

With this regard, the schools and teachers were the major source of SRH information for them. Teachers explained girls about menstruation hygiene and mostly promoted abstinence:

“*… also teachers always teach us how to use pads while having periods.”* (15 years old).

“*… .at school they* [teachers] *taught us to abstain from sex until we finish our studies and get married.”* (19 years old).

While did sometimes discuss other SRH topics, such as condom use and STIs, coverage of these topics was seen as poor or unsatisfactory by the students. One of the participants in the interview also mentioned that she learned about contraception methods by overhearing the women in the neighbourhood talking about them.

Access to health services among refugee girls was also found to be low with a total of 68.8% of participants who had never visited health centres to seek SRH services. Among the remaining 31.2%, the reasons for a visit were mostly HIV testing (22.7%) and menstrual problems (20.2%). About 83% of girls who ever visited a health care facility were willing to come back in the future. Some girls who did not want to return to the facilities pointed out the lack of privacy and mentioned the lack of resources, e.g. medicines in the centres due to which their problems could not be solved by the health workers. The girls also mentioned distance to the centres and mistreatment (e.g. judgments and impoliteness) by the health personnel as reasons for non-return.

When discussing the access to SRH services during qualitative interviews, the majority of girls did not know where to seek care related to SRH and were not aware about the location of the health centres. This could also be a reason for low number of visits to the SRH services. Interviewed girls who have already been living in the camp for longer had more knowledge about the existence of such services. Even then, they mentioned the distance to the facilities, lack of privacy and lack of health personnel as barriers to seek care for SRH problems. The issue of privacy was not only relevant to the health centres but also to schools, e.g. when girls wanted to discuss SRH aspects with teachers they were discouraged to do so knowing that they might share this information with others:

“*… .some of them* [teachers] *are tough. Some of them* [teachers] *when you tell them some problems of yours they also go to speak with fellow teachers.”* (16 years old).

### Sexual experiences including pregnancies and forced intercourse

Out of the total 260 female adolescents, 30 reported to be sexually active. The mean age for having the first sexual intercourse was 16 years (95% CI: 15.2, 16.8; SD 0.3). Among the sexually active girls, some (3) had intercourse between the age of nine and 12. A total of 36.6% of sexually active girls had forced intercourse and 23.4% of them had transactional intercourse. For the majority of the girls (46.7%), the partner was 1–5 years older. About 6.7% of them even had partners who were 10 or more years older than they were.

Among the 30 sexually active girls, a total of 70% of girls reported not using a condom the first time they had sexual intercourse. About 6.7% of them had used pills and the rest did not use any methods of contraception. A total of 46.7% of sexually active girls had been pregnant and for all of them, the outcome was a live child. But the pregnancy was desired only for 42.8% of those pregnant girls. The sexual experiences of the girls have been summarized in Table 4.

**Table 4:** Reported sexual experiences

	Number	Percentage
Ever had sex (*n* = 260):		
Yes	30	11.5
No	230	88.5
Mean age (*n* = 30):	16	NA
Forced intercourse (*n* = 30):		
Yes	11	36.6
No	19	63.4
Transactional sexual intercourse (*n* = 30):		
Yes	7	23.4
No	23	76.6
Pregnancy (*n* = 30):		
Yes	14	46.7
No	16	53.3
Was the pregnancy desired (*n* = 14):		
Yes	6	42.8
No	8	57.2
Use of condom at first intercourse (*n* = 30):		
Yes	8	26.4
No	21	70.0
Don’t know	1	3.6

When the sexual experiences in the past 3 months were considered, only 36.6% of girls were sexually active according to the survey. Among them 90.9% had single partner. The rest could not recall the number of partners. For 81.8% of them, the partner was a steady partner (boyfriend). However, only 18% of them reported using condoms during the last 3 months. About 63.6% of them did not know the HIV status of their partners and the rest were confident about the partner being HIV negative. Although not used, 54.6% of girls said to have discussed contraception with the last partner.

During the interviews, the participants often mentioned the fear of getting pregnant, as pregnancy before marriage was unacceptable in the society. This would cause a girl to be excluded from the community and even get expelled from school. This was a major factor that lead girls to abstain from sexual intercourse:

“*… some people in our community just chase a girl who becomes pregnant out of her home even if the parents are not aware of the issue …*” (16 years old).

“*… she was dismissed from boarding section [at school] … they expelled her.”* (16 years old).

The girls in the refugee camp were also victims of forced sexual intercourse. As discussed in the interview, some were victims of sexual violence during the war in their home country, some of them were raped in the camp itself and some on the migration way to the camp:

*“In Burundi there was a war, they would come and find you in the house, they mistreat [rape] girls and ladies and leave your father observing and later kill you … on the way we met people who wanted to rape us* …” (19 years old).

“*… on the way, we met thugs, they run after us and raped all of us … I came here with the pregnancy of my child which I acquired from Congo through rape.”* (19 years old).

During the interviews, it was found that not only forced sex but also coerced sex was prevalent in the camp:

“*… I had nothing to do, the man was very strong and older than me.”* (18 years old).

“*… men come to trick us and lie that they love us and may end up impregnating us.”* (19 years old).

Transactional sex, which was prevalent among adolescent refugees, was also discussed in school as mentioned by a participant during the interviews. She mentioned that in school they are made aware that they should avoid transactional sex:

*“We learn that students should avoid free gifts from men.* ” (17 years old).

Not a majority of them used condoms or other contraceptives the first time they had sexual intercourse. This was, as stated by the participants during the interviews, because of the fear of the partner or because the partner forced them to bear a child:

“*… I feared him and I didn’t use any contraceptives to stop pregnancy because of the fear.”* (18 years old).

### Female genital mutilation

Female genital mutilation (FGM) was seen to be prevalent in this population. About 10% of the participants of the study had been circumcised. They were mostly from Somalia (21), DR Congo (3), Uganda and Rwanda (3). The mean age when the circumcision took place was 7.3 (95% CI: 5.4, 9.1). For 44.4% of them, their mother made the decision that they would be circumcised. Some of them even reported that the decision was made by a close friend (7.4%).

The interviewers also talked to the girls about their experiences with FGM. The girls could relate very little of their experiences since for the majority, it took place when they were very young. But the consequence of the circumcision lies with the girls and they are constantly being affected by it. They complained of severe problems and pain during their menstruation. Not only the problems during menstruation, but some of adolescents also have difficulty with urination because of the circumcision. Despite all these difficulties, some of girls accept FGM as a part of their tradition:

“*…while in the menstruation periods they make me feel pain. I can urinate little, little not very well … but I am happy with it because all my friends, my mother, my sister, my aunts, we are all the same. So I don’t feel like it’s only me.”* (18 years old).

## Discussion

This study evaluated SRH knowledge, information sources and access to SRH services, existing sexual behaviour practices and experience of sexual violence among adolescent girls in a humanitarian setting in Uganda. Findings indicate that adolescent girls had limited comprehensive knowledge on SRH issues. Furthermore, school facilities offer an important source of SRH information for adolescents, however parents are a preferred source for these adolescents. Access to SRH services at health facilities are limited due to unfriendly services or lack of confidentiality. A few adolescents were sexually active (11.5%) and about 4.5% of girls overall had experienced sexual violence during the migration and settlement into the camp, describing situations that were unavoidable such as mass rape and “rite of passage”. Few sexually active adolescents had risky sexual practices such as limited condom use and transactional sex, in case of consensual sexual intercourse.

The overall SRH knowledge apart from HIV was low. Compared to adolescent nationals of a slightly younger age group in a recent study in South Western Uganda, adolescents in this study demonstrate low scores on knowledge of at least one effective method of pregnancy prevention - 44% vs 55%, knowing at least one STI - 52% vs 95%; however, knowledge of at least one way of HIV transmission was higher - 91.2% vs 47% respectively [21]. A related study in the same study area by Harrison et al. 2009 that compared refugees to nationals, established similar knowledge levels of HIV among 15–24 years old - 33.5% that were in comparable range to the national estimates [22]. A recent review also described limited SRH knowledge among adolescents in humanitarian settings in Sub-Saharan Africa [6]. Interventions are needed to enhance the knowledge and understanding of SRH issues within the refugee population to ensure these adolescents are on par with in-country counterparts.

Important sources of information on SRH for these adolescent girls were school and teachers. However, adolescents pointed out a few inadequacies related to the range of SRH topics taught, which were usually limited to abstinence with very little information on contraceptives. Adolescents also described constraints such as lack of trust or confidentiality of information shared with school teachers. The current enrolment of girls in schools, although satisfactory (63.4%), was lower in comparison to the findings of a study in a similar humanitarian setting in Somalia, where 91.4% of the adolescents (male and female) were currently enrolled in school [23]. There could be gender differences in these studies resulting from poor menstrual health management, forced and coerced intercourse resulted in pregnancy, contributing to school dropout among female adolescents. However, school sources offer good opportunities for sexuality education and a more comprehensive approach, e.g. recommended by UNESCO, to increase the coverage of SRH topics should be adopted in order to realise better outcomes [24]. Furthermore, parents were named as a preferred source for SRH information and similar studies described parents as important facilitators for behavioural changes and better SRH outcomes for adolescents [25]. Although adolescents shared that they are shy to discuss a broad range of SRH topics (usually limited to menstruation management) such social constraints of adolescent-parent communication in Sub-Saharan Africa has been identified before including inadequate knowledge, skills and cultural taboos. To enhance this communication, there is a need for strengthening of this important source of SRH information and behavioural change [26].

Furthermore, health centres were underutilised sources in this study, where 7.6% of girls sought SRH related information, yet only approximately 2.5% of them obtained contraceptive methods. This is low compared to an estimate by UNHCR of 58% of women who obtain family planning services from the SRH centres [13]. Although the results of the UNCHR report are from non-segregated data, the finding that access of adolescents to SHR services is generally limited has been described before [27]. Constraints to access SRH services at health facilities identified in our study were related to lack of knowledge about their location, fear of being judged/victimised by society and poor attitude of health workers - unfriendly or even rude.

The low use of contraceptives as found in our study (around 30% of sexually active girls ever used contraceptives at first sexual intercourse) corresponds to the findings reported in the UNHCR report in 2011, where around 40% reported the use of contraceptives [13]. Similar findings were reported by the study in Northern Uganda where less than 40% used condoms the first time they had sexual intercourse [28]. Eighteen percent of adolescents in our study used a condom the last time they had sexual intercourse, which was very similar to the results of the study by Casey et al., 2006 in Sierra Leone where 16% of adolescents used condoms the last time, they had sexual intercourse [27]. A study by Okanlawon et al., 2010 in Nigeria with a similar setting showed a higher proportion - 32.8% [29]. This study illustrates stagnancy in utilisation of contraceptive services in the settlement camp in Nakivale, and it has not progressed much comparing to the similar settings in 2006.

International guidelines emphasize prioritisation of provision of SRH information and services in humanitarian settings including vulnerable adolescents [30]. Operationalisation of these guidelines require a multi-pronged approach linking school-based sexuality education initiatives with parental support as well as linkage of adolescents to a wide range of SRH services, such as antenatal care, emergency contraception, services for victims of gender-based and sexual violence at health centres. It is important to address gender inequalities and marginalisation among refugees with particular attention to addressing harmful cultural practices such as FGM, gender based sexual violence and promoting access to contraception information and services among this vulnerable group.

Sexual activity was surprisingly low at only 11% in our study. A study in a transit camp in Northern Uganda has reported that 30% of women were being involved in sexual activity before the age of 15 [28]. This might be due to social desirability bias which plays a role in the underreporting of the sexual activity. The mean age for having the first sexual intercourse was 16, however, among those with consensual sex, at least 23% reported having had transactional sex, and 70% practiced sex without condoms. Earlier studies in the same setting showed that refugees were more likely to practice risky sexual practices compared to nationals [22]. Reasons for no condom use among girls in our study were related to fear of their partner or lack of knowledge of the contraceptive methods.

Adolescent girls also reported cases of forced and transactional sexual intercourse. Such cases were also described by Iyakaremye et al., 2016 in their qualitative findings in a refugee camp in Rwanda [31]. The proportion in our study was higher - 36.6% forced intercourse and 23.4% transactional intercourse among sexually active girls, than that reported in the study by Harrison et al., 2006 performed in Uganda – 5 and 10% accordingly [22].

### Study limitations

The study of sensitive SRH topics among adolescents has a lot of methodological challenges. The sampling method incorporated by the quantitative part of the study was a convenience sampling. It is easy and affordable sampling technic commonly used in research. However, it is likely to be biased, e.g. girls motivated towards or willing to obtain more information about SRH may have taken part in the interviews, which might sometimes not reflect the complete picture or miss hard-to-reach adolescents. Nevertheless, by targeting different communities and schools within the camp we tried to minimize potential bias. The attempt of the study was to include only the adolescent girls age 13–19 from the refugee camp. This was challenging as the reported age could not always be confirmed by valid documents for all cases. Because of this the study could have reporting bias in terms of age. Majority of the participants did not have knowledge of English; hence, an orally translated questionnaire was used. Nevertheless, questions which were back-translated may not always convey the exact and culturally adapted message. Underreporting is always a challenge when the study includes sensitive questions like sexual activity and contraception. Sexual activity was reported by very few participants. This might not reflect the real situation in the camp. Similarly, the girls might also have underreported transactional sex or sexual violence by their family members which is prevalent in studies in similar humanitarian setting. Thus, this study is not free from social desirability bias.

## Conclusions

Adolescent girls in humanitarian settings such as Nakivale settlement remain with limited SRH knowledge and access to SRH services. Schools and parents offer important information sources, though limited in coverage and quality. A multi-sectoral approach incorporating provision of comprehensive sexuality education for adolescents in schools and out of schools, with family support and facilitating access to youth friendly services including SRH commodities at health centres is important to reduce potential vulnerability of this group. A longitudinal intervention study to assess effectiveness of targeted SRH educational interventions on utilisation of SRH services and sexual behaviours in this vulnerable group would be helpful. Although a few adolescents were sexually active in this study, it’s important to recognise and mitigate emerging effects of sexual violence and risky sexual practices by offering counselling and rehabilitation of adolescents who are victims of forced sexual intercourse during transit or within the camp.

## Additional file

Additional file 1: Tabulated results from the multinomial regression analysis for each of the individual covariates and the corresponding Relative Risk Ratio’s (RRR), Confidence Intervals (CI) and *p*-values. (DOCX 16 kb)

## Abbreviations

AIDS: Acquired Immune Deficiency Syndrome
CDC: Centre for Disease Control and Prevention
FGD: Focused Group Discussion
HIV: Human Immunodeficiency Virus
IC: Informed Consent
NGO: Non-Governmental Organization
SRH: Sexual and Reproductive Health
STI: Sexually Transmitted Infection
UN: United Nations
UNFPA: United Nations Population Fund
UNHCR: United Nations High Commission for Refugees
