# Supplementary material for: A cross-sectional mixed-methods study of sexual and reproductive health knowledge, experiences and access to services among refugee adolescent girls in the Nakivale refugee settlement, Uganda
Source: Reprod Health. 2019 Mar 19;16:35. doi: 10.1186/s12978-019-0698-5 (PMC6425697; doi:10.1186/s12978-019-0698-5)
Supplement: Supplementary file 1 — Tabulated results from the multinomial regression analysis for each of the individual covariates and the corresponding Relative Risk Ratio’s (RRR), Confidence Intervals (CI) and p-values. (DOCX 16 kb) [file 12978_2019_698_MOESM1_ESM.docx]

**Additional file 1**

**Tabulated results from the multinomial regression analysis for each of the individual covariates and the corresponding Relative Risk Ratio’s (RRR), Confidence Intervals (CI) and p-values.**

|  | | *Outcome of overall knowledge*: Comparator **Low** | | | | | |
| --- | --- | --- | --- | --- | --- | --- | --- |
|  |  | **Medium** | | | **High** | | |
| *Covariate* | *Comparator* | *RRR* | *CI* | *p value* | *RRR* | *CI* | *p value* |
| **Age group**  (>15 years) | <=15 years | 1.20 | (0.68,2.10) | 0.53 | **2.78** | **(1.29,5.98)** | **0.01** |
| **Age** (continuous) | | 1.14 | (0.97,1.33) | 0.12 | **1.38** | **(1.12,1.69)** | **<0.01** |
| **Living in Uganda** | | | | | | | |
| 3-5 years | < 3 years | 0.72 | (0.34,1.51) | 0.39 | 1.13 | (0.42,3.09) | 0.80 |
| > 5 years | < 3 years | 1.10 | (0.57,2.12) | 0.77 | 1.94 | (0.82,4.61) | 0.13 |
| **Education** |  |  | | | | | |
| Secondary/  Tertiary | Primary | **8.21** | **(2.43,27.79)** | **<0.01** | **45.92** | **(12.55,167.94)** | **<0.01** |
| **Religion** | | | | | | | |
| Catholic | Protestant | 0.69 | (0.32,1.51) | 0.36 | 1.08 | (0.69,4.10) | 0.25 |
| Others | Protestant | 1.69 | (0.51,2.28) | 0.85 | 1.26 | (0.48,3.32) | 0.65 |
| **In School** | | | | | | | |
| No | Yes | 0.76 | (0.43,1.35) | 0.35 | 0.61 | (0.28,1.30) | 0.20 |
| **Ever had sex** | | | | | | | |
| No | Yes | 0.30 | (0.10,0.91) | 0.03 | 0.28 | (0.08,1.00) | 0.05 |
| **Country of birth** | | | | | | | |
| Burundi/  Rwanda | DR Congo | 1.97 | (0.94,4.12) | 0.07 | 0.97 | (0.37,2.49) | 0.94 |
| Eritrea/  Ethiopia | DR Congo | 0.66 | (0.27,1.63) | 0.37 | 0.71 | (0.24,2.11) | 0.54 |
| Somalia/  South Sudan | DR Congo | 1.61 | (0.63,4.15) | 0.32 | 1.70 | (0.57,5.05) | 0.34 |
| Others | DR Congo | 2.18 | (0.80,5.94) | 0.13 | 0.66 | (0.15,2.89) | 0.58 |
| **Main source of knowledge** | | | | | | | |
| School Teachers | Parents | **2.60** | **(1.27,5.34)** | **0.01** | **8.80** | **(3.40,22.80)** | **<0.01** |
| Others | Parents | 1.05 | (0.54,2.05) | 0.89 | 1.12 | (0.37,3.40) | 0.84 |

The outcome is obtained as a categorical classification of low, medium and high, based on the sum of the scores from the knowledge on prevention measures and transmission methods for HIV/AIDS as well as knowledge on STIs and methods of contraception (see Table 3 in the manuscript). The statistically significant covariates or covariate categories at 5 % level of significance are shown in bold values of RR, CI and p value.
